# Current standards and ethical landscape of engineered tissues—3D bioprinting perspective

**DOI:** 10.1177/20417314211027677

**Published:** 2021-07-29

**Authors:** Muthu Parkkavi Sekar, Harshavardhan Budharaju, Allen Zennifer, Swaminathan Sethuraman, Niki Vermeulen, Dhakshinamoorthy Sundaramurthi, Deepak M Kalaskar

**Affiliations:** 1 Tissue Engineering & Additive Manufacturing Lab, Centre for Nanotechnology & Advanced Biomaterials, ABCDE Innovation Centre, School of Chemical & Biotechnology, SASTRA Deemed University, Thanjavur, Tamil Nadu, India; 2 Department of Science, Technology and Innovation Studies, School of Social and Political Science, University of Edinburgh, High School Yards, Edinburgh, UK; 3 UCL Division of Surgery and Interventional Science, London, UK

**Keywords:** TEMPs, regulations & standards, 3D bioprinting, stem cells, ethical concerns, tissue engineering

## Abstract

Tissue engineering is an evolving multi-disciplinary field with cutting-edge technologies and innovative scientific perceptions that promise functional regeneration of damaged tissues/organs. Tissue engineered medical products (TEMPs) are biomaterial-cell products or a cell-drug combination which is injected, implanted or topically applied in the course of a therapeutic or diagnostic procedure. Current tissue engineering strategies aim at 3D printing/bioprinting that uses cells and polymers to construct living tissues/organs in a layer-by-layer fashion with high 3D precision. However, unlike conventional drugs or therapeutics, TEMPs and 3D bioprinted tissues are novel therapeutics and need different regulatory protocols for clinical trials and commercialization processes. Therefore, it is essential to understand the complexity of raw materials, cellular components, and manufacturing procedures to establish standards that can help to translate these products from bench to bedside. These complexities are reflected in the regulations and standards that are globally in practice to prevent any compromise or undue risks to patients. This review comprehensively describes the current legislations, standards for TEMPs with a special emphasis on 3D bioprinted tissues. Based on these overviews, challenges in the clinical translation of TEMPs & 3D bioprinted tissues/organs along with their ethical concerns and future perspectives are discussed.

## Introduction

Tissue engineered products including naturally derived or synthetic biomaterial-based scaffolds with/without autologous or allogeneic cells are utilized to replace or restore the functions of damaged tissues and organs.^1^ Advanced therapy medicinal products (ATMPs) are considered as medicines for the treatment of disease or injuries in humans using genes, tissues or cells. ATMPs are classified into categories such as gene therapy medicinal products (GTMP), cell therapy medicinal products (CTMP), tissue-engineered medicinal products (TEMPs) and sometimes a combination of these categories.^2^ In some countries like the US, a group of biological medicinal products are categorized as Human Cells, Tissues, and Cellular and Tissue-based Product (HCT/P).^1,3^ ATMPs have several applications in the clinical arena to improve the quality of patients life. Organ transplantation is the terminal-stage treatment strategy for conditions like myocardial infarction, acute liver failure, chronic kidney disease, and Type 1 diabetes, etc. and may replace the need for TEMPs^4^ However, shortage of organ donors, high cost and immunological complications limit the affordability and clinical success of organ transplantation around the globe.^5,6^ Thus, there is a need for artificial organ fabrication through tissue engineering approaches to overcome the limitations of organ transplantation.^7^ Although various approaches have been used to develop engineered tissues, only a limited number of TEMPs are approved and commercialized for clinical uses (Table 1). This may be attributed to the fact that engineered tissues have high risks, more uncertainties in safety, efficacy and very expensive for commercialization.^8^

**Table 1.** List of approved tissue engineered medical products (TEMPs) commercialized in various countries.

Product name	Description	Intended application	Year of commercialization	Origin
Omnigraft^™^	Bilayer graft comprising upper silicone layer and lower collagen and chondroitin layer for chronic diabetic foot ulcers	Skin^1,3,9,10^	2016	US (United States)
ReNovaCell^™^	Skin autologous (epithelial cell) harvesting device as skin graft for vitiligo		2016	Europe
Hyalograft 3D^™^	Cultured autologous (skin fibroblast) on hyaluronic derivative scaffold for diabetic foot ulcer		2007	South Korea
Dermagraft^®^	Cultured neonatal dermal fibroblast on bioresorbable scaffolds for foot ulcer treatment		2001	US
Ossron^™^	Autologous (bone marrow stem) implantation-based cell therapy for new bone formation	Bone^1^	2009 2017	South Korea, India
INFUSE^®^ bone graft	Recombinant human bone morphometric protein—2 on an absorbable collagen sponge for bone grafting (spine and orthopedic)		2015	US
MACI^®^	Cultured autologous cell source (chondrocytes) on porcine collagen scaffold for treating damaged cartilage tissue	Cartilage^1,11,12^	2016	US
Ortho-ACI^™^	Autologous chondrocytes seeded with the scaffold for cartilage defect		2017	Australia
Spherox	Human autologous (chondrocyte) spheroids for cartilage defect in adults		2007 2017	Germany Europe
JACC^®^	Cultured autologous (chondrocytes) cells on collagen for cartilage defect		2012	Japan
Novocart 3D	Autologous chondrocytes on 3D collagen chondroitin sulfate scaffold for cartilage defect		2003	EU
CardioCel^®^	Acellular collagen matrix-based scaffold for cardiovascular treatment	Heart^1,13^	2013 2014 2014 2015	Europe US Canada Singapore
HeartSheet^®^	Autologous skeletal myoblast derived cellular sheets for ischemic heart disease		2015	Japan
Avance^®^ nerve graft	Decellularized ECM based 3D scaffold (allograft) for bridging nerve gap	Nerve^14^	2015	US
Neurotube^®^	Polyglycolic acid (PGA) based mesh tube for small pheripheral nerve lesions		1999	US

TEMPs are the latest therapeutics that involve one or more complex manufacturing processes, varied constituents and different characteristic features, which demands unique standards and regulations for approval processes.^15^ Global regulatory agencies evaluate the quality, safety, efficacy and cost-effectiveness of TEMPs for healthcare applications and regulate through various steps involved from commercialization to clinical practice.^8,16^ Tissue engineered medical products are regulated based on exclusively framed legislations in different countries. These legislations have the same set of objectives and rules but follow different regulatory terms/approval processes for commercialization.^15^

TEMPs submitted under clinical transformation approval need to be manufactured in Good Manufacturing Facilities (GMP) setup using clinical-grade raw materials with defined Quality Attributes (QAs) and also need to be tested clinically with Good Clinical Practice (GCP) guidance.^17^ Regulatory authorities of medicinal product approval generally focus on the criteria mentioned above and more specifically, on its benefits and risk involvement.^8^

**Figure 1.**
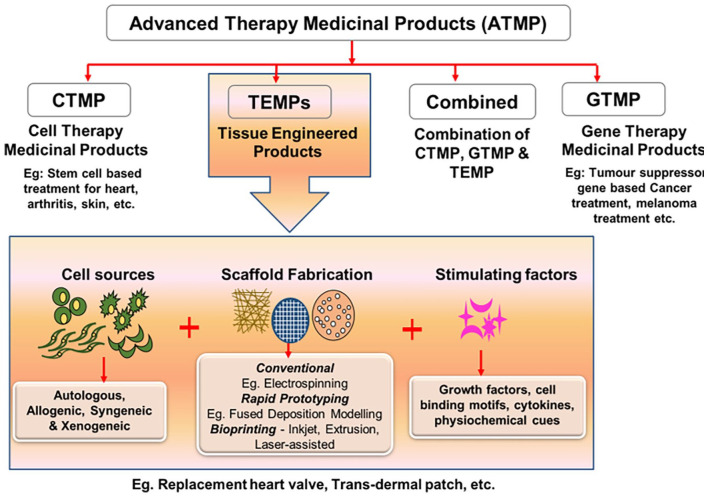
Schematic representation of components and classification of ATMPs and their applications.

**Figure 2.**
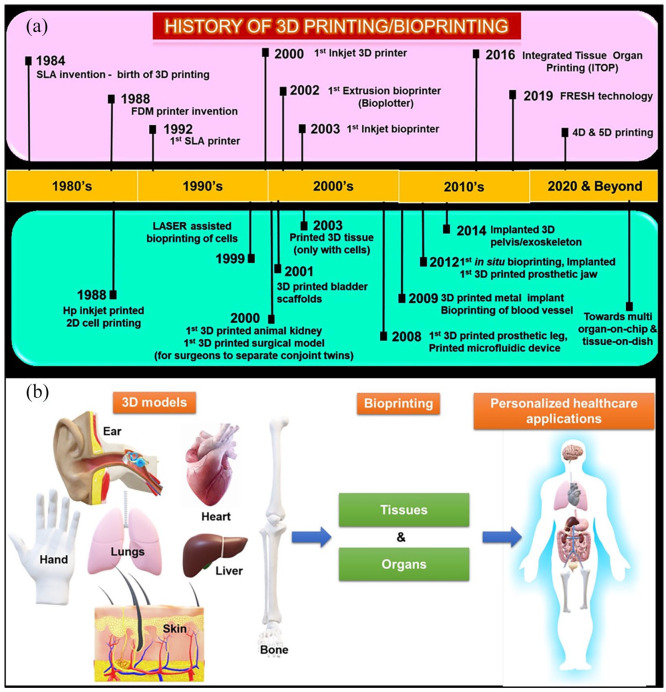
(a) Chronological history of 3D printing/bioprinting in potential biomedical applications, (b) imagined future of 3D bioprinting—3D models obtained through CT or MRI images and computationally redesigned according to the personalized requirements.

In the current decade, TEMPs fabricated through conventional tissue engineering approaches have a wide range of potential applications, such as congenital heart disease (CHD), heart valve replacement, bone fracture healing, severe burn injuries, cartilage defect, acute/chronic liver problems, etc., are now considered as a safer and efficient clinical solution.^4^ Figure 1 shows the classification of ATMPs with emphasis on TEMPs, which falls under the main scope of this review. Further, bottom-up and reverse engineering approaches for fabricating patient-specific three-dimensional cellular/biomaterial scaffold with autologous cells (stem cells/other cells) using 3D bioprinting technique has become a better strategy in fabricating organs with biomimetic geometries and other physiological features of native tissues without any immune complications.^15,16,18–20^ 3D printing has greatly evolved as an efficient technology especially for various applications in regenerative medicine. The progress and applications of 3D printing and bioprinting in regenerative medicine were broadly represented in Figure 2(a). Hence, computerized fabrication of multi-functional human 3D organs could facilitate the ease of organ transplantation and 3D tissue/organ model fabrication for pre-clinical drug/biological testing in the future (Figure 2(b)).

From the above perspectives, this review mainly focuses on all the legislations, regulations, guidelines and standards followed worldwide for clinical trial approval and commercialization of the tissue engineered products. Further, the current tissue fabrication process with the advancement of additive manufacturing approaches for engineering personalized 3D printed tissues, components & types of 3D bioprinting, and the potential of bioprinting technology to fabricate tissues/organs for transplantation applications are also discussed. Additionally, the characteristics of bioprinted constructs, their applications and how they stand in comparison with tissue engineered medical products from an ethical and regulatory standpoint are elaborated. Further insights into the regulatory aspects of tissue engineered and bioprinted organs/tissues that greatly hamper the widespread clinical translations are discussed. Additionally, the ethical issues revolving around tissue engineered and bioprinted products and challenges in commercial success are also discussed. Finally, future perspectives of tissue engineered constructs are discussed and compared with the projected future of 3D bioprinting in regenerative medicine applications.

## Tissue engineered medical products (TEMPS)

Tissue engineering (TE) is an interdisciplinary field that employs the principles of life sciences and engineering to restore and regenerate the physiological activity of damaged tissues or organs. This has led to the rapid development of tissue engineered medical products (TEMPs) using conventional tissue engineering strategies for diagnostic and therapeutic applications in various tissues such as skin, cartilage, bone, blood vessels, heart valves, etc., using components such as cells, scaffolds, biomolecules, processed tissues and their derivatives.^21^ Notably, there are several TEMPs for cartilage defects and skin substituents which are currently in phase II and III clinical trial stages and so far provided the confirmations on therapeutic value, regenerative activity, safety and long-term biological effects.^22,23^ Moreover, the increase in clinical demands for organ transplantation, specifically for aging populations has propelled the exponential need for TEMPs on a larger scale and at affordable prices. However, limitations in conventional fabrication techniques such as lack of three-dimensional architecture, cellular positioning at the desired locations, variable cellular density, template requirement, difficulty in complex shape fabrication suggest the usage of advanced fabrication techniques to engineer tissues or organs for transplantation applications.^24,25^

### An additive manufacturing approach: 3D printed TEMPS

3D printing has now gained huge attention from many users of different fields due to the layer-by-layer deposition, feasibility in fabricating complex models, ease of operation, low cost and multi-material deposition.^26^ Various 3D printing techniques have been employed to develop patient-specific scaffolds and tissues using polymers such as polylactic acid (PLA), titanium, ceramics, polycaprolactone (PCL), polyurethane, etc. and most of them are evaluated for durability and functionality in both in vitro and in vivo studies for tissues such as bone, heart valves, skin, intestines, etc.^27^ Currently in clinical surgery, 3D printing technology plays a major role by providing different technical and visual or physical support in the form of pre-operative surgical guides (cutting/drilling/planning), surgical tools, custom specific implants and prostheses.^28^ 3D printing offers a wide range of materials selection approach to create 3D structures as support or major implant for spinal surgery, maxillofacial, cranial, dental, orthopedic surgery, etc. Undoubtedly, for all these surgical approaches, medical surgeons collaborate with additive manufacturing based medical device companies or bioengineers to assist in the design and 3D printing for specific requirements. Additionally, 3D planning software makes patient-specific virtual surgery model to provide the clear-cutting plane and drilling trajectories to limit damages for nerves and blood vessels and improves positioning accuracy to place the implant.^29^ Subsequently, the printing of different sized models can help to overcome the limitation of using cadaver-based surgical planning where specificity and availability are a major issue.^30^

In a recent study of tissue regeneration to mimic native tissue structure, 3D printed bone graft made from PCL impregnated chitosan loaded with rabbit bone marrow stem cells showed improved differentiation activity and increased expression of osteogenic specific genes such as alkaline phosphatase (ALP), collagen type I (COL1), osteocalcin (OCN), and Runt-related transcription factor (RUNX2) after 14 days in vitro. This graft was also subcutaneously implanted in nude mice and observed that the 3D printed scaffold exhibited stronger osteogenesis and bone-matrix formation after 3 weeks of surgery.^31^ Similarly, Zhang et al. have developed 3D printed navigational template using Computer Aided Design (CAD) software to facilitate the localization of small peripheral lung nodules and reduces the radiation exposure for lung cancer patients (ID: NCT02952261). The results of about 200 patients demonstrated that the personalized 3D printed template guided percutaneous localization was achieved with better accuracy and omits computed tomographic (CT) analysis during treatment.^32^

### 3D bioprinting of tissues and organs

**Figure 3.**
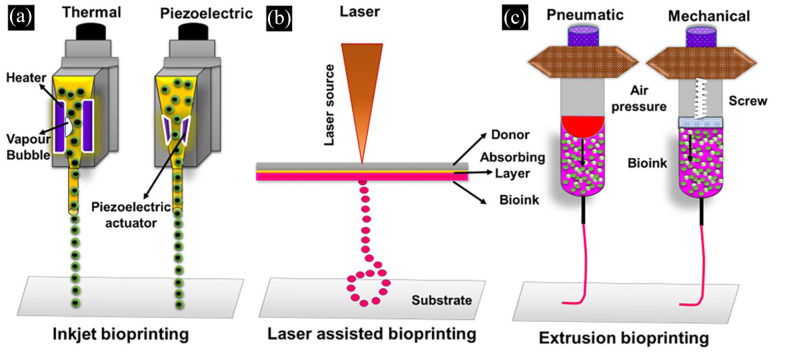
Schematic representation of different types of bioprinting. (a) Inkjet bioprinting, (b) laser-assisted bioprinting and (c) extrusion bioprinting.

3D bioprinting technique is a 3D tissue fabrication technique where the cells are integrated into a cross-linkable hydrogel matrix called bioink to create 3D tissue equivalent constructs in the desired pattern.^33,34^ 3D bioprinting requires essential components such as 3D imaging, CAD/CAM software, bioink, and bioprinter to carry out the fabrication process. Bioink is a combination of cells, polymers (biomaterials) and signaling molecules like growth factors with adequate viscoelastic and cell supportive functionalities that are suitable for the printing of tissues and organs in a bioprinter platform.^35^ Further, the development of novel biomaterials or chemical modification of existing materials helps to customize bioinks for tissue-specific applications. Moreover, bioinks are deposited in a layer-by-layer manner at desired locations to enable the fabrication of vascularized 3D structures for tissue or organ regeneration applications. Bioink made of fibrous proteins such as collagen and fibrin contains more than 90% water, allowing them to create cell supportive and biocompatible tissue constructs.^36^ Natural polymers such as collagen, Matrigel, gelatin, alginate, agarose, methylcellulose, fibrinogen and synthetic polymers such as pluronics, carbopol, nanoclay, hydroxyapatite and poly(*N*-isopropylacrylamide) have been used as bioinks for the fabrication of various tissues like cardiac patches, bone, cartilage, and cornea.^37^ Bioprinters capable of achieving ideal print speed, human-scale resolution, and also equipped to operate multi-material print heads could be beneficial in the fabrication of patient-specific tissues or organs. Bioprinting is divided into three types based on the bioink dispensing mechanism such as inkjet bioprinting, laser-assisted bioprinting and extrusion-based bioprinting. Inkjet bioprinting requires less viscous bioinks to dispense through micron-sized nozzles either by thermal or piezoelectric stimulus (Figure 3).^38^ Laser-assisted bioprinting methods employ focusing the laser pulse on the metal-coated plate with low—medium viscosity bioinks to create 3D tissues (Figure 3).^39^ These two printing systems have good printing resolution (20–40 µm) and cell viability (>90%). However, these methods are not widely preferred for fabricating human scale 3D tissues due to the difficulties in layer-by-layer stacking ability beyond a particular build volume. Extrusion bioprinters can dispense a variety of bioinks with a wide range of viscosities either through pneumatic or mechanical screw-based mechanisms (Figure 3). In recent years researchers have successfully fabricated various 3D tissues and organ models as a proof-of-concept using extrusion bioprinting.^40^

Further, the features and limitations of these various bioprinting techniques such as inkjet, extrusion and laser-assisted bioprinting methods are briefly tabulated in Table 2. Printing of multiple cell types with variable cell densities (1 × 10^6^ cells/mL to 1 × 10^8^ cells/mL) at desired locations made 3D bioprinting one of the most advanced techniques for tissue or organ fabrication.^41^ A comprehensive literature review of 3D tissues/organs fabricated using different bioprinting strategies for tissue regeneration application has been tabulated in Table 3.

**Table 2.** Comparison of different techniques utilized in 3D bioprinting of tissues.

	Inkjet bioprinting	Laser-assisted bioprinting	Extrusion bioprinting
Contact	Non-contact	Non-contact	Contact
Dispensing form	Droplets	Droplets/continuous deposition	Filament
Dispensing mechanism	Thermal and piezoelectric	Laser	Pneumatic or mechanical
Printing speed	1–10,000 droplets/s	1–2000 mm/s	0.1–150 mm/s
Viscosity	<10 mPa s	1–300 mPa s	30–1 × 10^6^ mPa s
Resolution	20–100 µm	40–100 µm	40–1200 µm
Materials used as bioinks	Alginate, gelatin, collagen type I, fibrin, polyethylene glycol (PEG) and gelatin methacrylate, etc.	Alginate, collagen type 1, gelatin, fibrin, etc.	Alginate, gelatin, gellan gum, guar gum, methylcellulose, collagen type 1, matrigel, fibrinogen, collagen methacrylate, gelatin methacrylate, elastin, polycaprolactone, polyethylene glycol, polyvinyl alcohol, polyvinyl acetate, etc.
Cell viability	⩾85%	⩾90%	60%–90%
Fabrication level	Cells and tissues	Cells and tissues	Cells, tissues and organs
Printed tissues	Cardiac, liver, muscle, and bladder	Bone, cardiac, skin, cornea and nerve, etc.	Cardiac, pancreas, bone, skin, intestine, liver, kidney, and nerve, etc.
Advantages	Fast and cost-effective	Cytocompatibility, single-cell and multiple cell types printing	Printing of viscous materials, the printing of multiple cell types, and ease of 3D structures fabrication
Disadvantages	Clogging, non-uniformity in droplet size and difficulty to dispense viscous materials	Low efficient to form 3D structures, unable to dispense viscous materials and affordability	Low resolution, needle clogging and shear-induced cell death
References	Derakhshanfar et al.^42^ and Ding et al.^43^	Koch et al.^44^ and Guillotin et al.^45^	Lee et al.^46^ and Gaetani et al.^47^

**Table 3.** Various types of tissue/organ constructs fabricated using 3D bioprinting methods.

Tissues/organs	Bioink	Printing method/printer type	Cell’s type	Cell viability	Significant observations	References
Nerve	Alginate, RGD—alginate, hyaluronic acid (HA), fibrin	Extrusion bioprinting (3D bio-plotter, EnvisionTEC)	Schwann cells from the sciatic nerve	⩾89%	Schwann cell incorporated constructs showed good viability, elongation and directional growth of neurites in the longitudinal axis of strands and supported peripheral nerve regeneration after injury	Ning et al.^48^
Nerve	Gelatin methacrylate (GelMA)	Digital light processing (DLP)-printer	Schwann cells (S16) and human umbilical vein endothelial cells (HUVECs)	–	Nanoparticles-in-hydrogel nerve conduit that releases drug to facilitate peripheral nerve regeneration via Hippo pathway	Tao et al.^49^
Intestine	Collagen type-I	Vertically moved 3D printing	Caco-2 cells	90%	Cell laden collagen/SIS villi showed significant cell proliferation, glucose uptake, tight-junction proteins and permeability coefficient	Kim and Kim^50^
Skeletal muscle	Fibrinogen, gelatin, HA, glycerol, polycapro-lactone (PCL)	Extrusion bioprinting (3D ITOP)	Human muscle progenitor cell (hMPC)	90%	Bioprinted skeletal muscle tissue exhibited organized multi-layered muscle bundles with aligned myofiber-like structures showed 82% of functional recovery in a rodent model 8 weeks of post-implantation	Kim et al.^51^
Skin	Collagen	Extrusion bioprinting (integrated composite tissue/organ building system (ICBS))	Human primary dermal fibroblasts (HDFs) and epidermal keratinocytes (HEKs)		The hybrid 3D cell printing system has extrusion and inkjet modules. Printed structures showed dermis and stratified epidermis layers after 14 days in vitro	Kim et al.^52^
Bone	Collagen and bioceramic (β-tricalcium phosphate)	Extrusion bioprinting (3-axis printing system)	Pre-osteoblast cells (MC3T3-E1) and human adipose stem cells (hASC)	⩾92%	The hASC-laden β-TCP composite structure showed significant osteogenic gene expression levels	Kim and Kim^53^
Bone	Endothelial cell growth medium	Laser-assisted bioprinting	Mesenchymal stem cells (MSC)	–	In situ printing of HUVECs improved vascularization and bone regeneration of critical sized bone defects in mice	Kérourédan et al.^54^
Cartilage	Nanofibrillated cellulose	Extrusion bioprinting (INKREDIBLE 3D Bioprinter)	Human chondrocytes and human MSC	–	Printed constructs displayed 17.2% surface area covered with proliferating chondrocytes after 60 days of culture in vitro	Apelgren et al.^55^
Liver	Lung derived human extracellular matrix (hECM), gelatin and sodium alginate	Extrusion bioprinting (INKREDIBLE+)	Human bipotent hepatic progenitor cells and human epithelial lung carcinoma cells	–	Alginate/gelatin bioink with hECM enhances cell viability and liver-specific metabolic activities	Hiller et al.^56^
Cardiac	GelMA, CdECM	Extrusion bioprinting (EnvisionTEC 3D-bioplotter developer series)	Neonatal human cardiac progenitor cells	75%	Printed GelMA patch with dECM have shown better proliferation, differentiation and angiogenic potential	Bejleri et al.^57^
Cardiac	Alginate Geltrex, and fibrin, HA	Laser-assisted bioprinting	hiPSC and iPSC—cardiomyocytes (iPSC-CM)	82%	Laser printing does not affect the pluripotency, differentiation and proliferation ability of iPSC	Chichkov et al.^58^
Cornea	Collagen type 1, hyaluronic acid, laminin	Laser-assisted bioprinting	Human embryonic stem cell-derived limbal epithelial stem cells and human adipose-derived stem cells (hASC)	–	Successfully used laser bioprinting for corneal applications using human stem cells and showed layered 3D bioprinted tissues mimicking the structure of native corneal tissues	Sorkio et al.^59^
Cornea	Gelatin, alginate and type 1 collagen	Extrusion bioprinting	Human corneal epithelial cells (HCECs)	⩾94%	The bioprinted cells showed good cell proliferation and expression of cytokeratin 3 (CK3) with the developed bioink of collagen, gelatin and alginate	Wu et al.^60^
Nose	Poly(ε-caprolactone) and gelatin	Extrusion bioprinting	hiPSC-derived mesenchymal stem cells (MSCs)	86%	This double gelation mechanism and developed constructs showed good layer stacking ability and it was able to print nose as proof of concept	Hsieh and Hsu^61^
Blood vessels	GelMA and alginate	Extrusion bioprinting (Novogen MMX bioprinter)	HUVECs and human mesenchymal stem cells (MSCs)	⩾80%	GelMA/alginate blended bioink exhibited good biological characteristics that supported the spreading and proliferation of encapsulated endothelial and stem cells	Jia et al.^62^
Blood vessels	Agarose and collagen type 1	Microvalve-based (drop-on-demand bioprinting)	Human dermal fibroblasts (HDF) and HUVECs	–	Agarose/collagen hydrogel blend can be 3D printed and printed constructs exhibit cell-induced vascularization capability	Kreimendahl et al.^63^

## Regulatory roles and responsibilities of approving authorities

The main goal of regulatory agencies is to produce laws and regulations by following different regulatory frameworks to allow safer medicinal products for clinical trials and marketing purposes. Globally tissue-based products (TEMPs) are identified differently based on the categorizing condition for the regulatory approval. Likewise, TEMPs have been considered as drugs in the EU, a medical device in Japan and biologic/combined products in the U.S through their regulations/directives and further their approval pathways are decided by assessing the quality, safety and efficacy through nonclinical and clinical studies.^3,12,64^ The regulatory agencies of different countries that deal with clinical trial approval and permission for commercialization are listed in Table 4.

**Table 4.** List of regulatory agencies authorized in various countries for medicinal product regulation.

Regulatory agency	Country
FDA’s (Food and Drug Administration)—established Center for Devices and Radiological Health (CDRH) for medical device regulation, Center for Biologics Evaluation and Research (CBER) and Center for Drug Evaluation and Research for drug regulation	USA
Pharmaceuticals and Medical Device Agency (PMDA) and Ministry of Health, Labor and Welfare (MHLW)	Japan
European Medicines Agency (EMA)	Europe
Ministry of Health and Family Welfare, Central Drug Standards Control Organization, Indian Council of Medical Research	India
Ministry of Health of the Russian Federation	Russia
State Food and Drug Administration (SFDA)	China
Department of Health, Health Protection Agency, Medicines and Healthcare Products Regulatory Agency	United Kingdom
Ministry of Health, National Institute of Pharmacy and Medicines, National Authority of Medicines and Health Products	Portugal
Ministry of Social Affairs and Health, Finish Medicines Agency	Finland
Korean Food and Drug Administration (KFDA)	South Korea

Identically, Gene and Cell-based Therapies (GCTs) regulatory frameworks are outlined to assess product characteristics, evidentiary and non-evidentiary reasons for approval and post-marketing risk management studies.^8^ It is identified that clinical translation of products is approved based on confirmed evidential benefit results for US and EU applications while regulations of Japan follow non-confirmed beneficial results. US, EU and Japan regulatory agencies are now approving Gene and Cell-based Therapies (GCTs) with scientific uncertainties and safety risks to allow the clinical transformation based on medical needs.^8^ Oftentimes, approval processes for new products are based on the standards available on the technical details of pre-existing relevant products.

PROVENGE was the first commercialized cell therapy treatment for prostate cancer treatment in the United States and Transcyte for third degree skin replacement graft was the first FDA approved tissue graft in 1997.^12^ Activskin, a skin graft was the first approved TEMP in China by 2013.^15^ In 2001, the first cell therapy and tissue engineered product (chondrocyte-based product) was successfully commercialized in South Korea. Relatively, South Korea has more number of cell therapies in clinical practice as on 2018 status.^1^ Totally 10 ATMPs were approved by the European Medicines Agency (EMA) in the Europe Union and likewise, the first commercialized tissue engineered product in Europe is ChondroCelect (for treating cartilage defects) in 2009, but this product was discontinued in 2016 due to commercial reason from its marketing authorization TiGenix NV.^12^ As discussed earlier, in comparison with the last decade, more tissue-based therapies are approved by the authorities in consideration to produce these high therapeutically efficient TEMPs for the unmet medical needs.^10^ As a result, several products for skin, cartilage, bone, heart, neural and several other complex organs have been commercialized in recent times.

### Current regulations for TEMPs

Recently updated tissue engineered product regulations, approval requirements and rules enforced so far in U.S, India and European Union for the currently available TEMPs are shown in Table 5.

**Table 5.** Comparison of current regulations followed by U.S., India and European Union for approval of clinical trials and commercialization of tissue engineered medical products (TEMPs).

	Food and Drug Administration (FDA)—US^65^		Central Drug Standards Control Organization (CDSCO)—India^66^		European Medicines Agency (EMA)—EU^67^	
Legislations related to the tissue engineered products commercialization for clinical practice in humans	FDA tired and risk based regulatory criteria for human cells, tissues, and cellular and tissue-based product (HCT/P) are followed by Centre for Biologics Evaluation and Research (CBER)		Import, manufacture, distribution and sale of drug and cosmetics in India tissue engineered products are also regulated by this act in consideration as therapeutic drugs		Advance therapy medicinal products (ATMPs) including gene, somatic therapies and tissue engineered product-based therapies and treatment enforced by committee for advanced therapies (CAT) and EU marketing authorization by regulating it as biological products	
	Regulated under	Objective	Regulated under	Objective	Regulated under	Objective
	21 CFR part 1271 and section 361 of the public health service act (PHS Act) (42 U.S.C. 264) (regulation for HCT/Ps found under criteria mentioned in 1271.10(a))	Establish the product requirements, details regarding registration, listing details, donor eligibility, good tissue practice (GTP), marketing details and steps/procedures to prevent the spread of communicable disease to patients No need of premarket approval	Drug and cosmetic act 1940 and Rules 1945 (Amendment, 2016)	Regulation of new drug/cosmetics, assist its manufacture, import and marketing in India with high standards and assured safety for human health care application through licensing. It also regulates Ayurvedic, siddha and Unani drug manufacture and import	REGULATION (EC) No C(2017) 7694	To regulate manufactured ATMP for clinical trials and exploring new possibilities of diagnosis and treatment in evaluation of its quality, safety and efficacy in order to provide approval for marketing in EU areas
	Section 351 of the PHS Act (42 U.S.C. 262) and/or federal food, drug, and cosmetic act (FD&C Act) (HCTPs regulation as other drugs, devices, and/or biological products)	Establish product requirements, details regarding biologics license (with respect to the product risk & safety analysis), clinical evaluation, post-market studies, labeling and marketing				
	Final rule	Scope	Rule (schedule Y)	Scope	Regulations/directive	Scope
Rules to be followed further	66 FR 5447, January 19, 2001 69 FR 3823, January 27, 2004	Registration and listing requirements and procedures	Rule 122 A	Application of permission to import new drug (licensing authority approval)	Article 11 of directive 2001/83/EC	Product characteristics and technical requirements to demonstrated product safety, quality and efficacy
	69 FR 29786, May 25, 2004 70 FR 29949, May 25, 2005 (Revised)	Cell source donor eligibility details and criteria (screening and testing mandatories)	Rule 122 DA	Requirement of permission from DCG(I) to conduct clinical trial for new drug/investigational new drug	Regulation (EC) No 726/2004	Centralized authorization procedure—scientific valuation of safety, efficacy and quality
			Rule 122 DAB	Provision of examining serious adverse event (SAE) of death/injury and compensation payment details and debarment in case of failure	Directive 2004/23/EC	Comprises standards of quality and safety for the donation, procurement, testing, processing, preservation, storage and distribution of human tissues and cells
	69 FR 68612, November 24, 2004	Includes the establishment, inspection of the product GCTP	Rule 122 DAC	Mandatory requirement of comprising good clinical practice (GCP) guidelines and specified rules in schedule Y of drug and cosmetics rules Provision of debarment of applicant in case of non-compliance	Regulation (EU) No 536/2014 and directive 2001/20/EC	Comprises principle and guidelines for clinical trials and good manufacturing practice applicable to investigational medicinal products
			Rule 122 DD	Registration of ethical committee to safety, rights and well beings of human subjects under clinical trial study	Directive 2005/28/EC directive 2003/94/EC	Good clinical practice good manufacturing practice

#### US based regulations

Unites States (US) authorized Food and Drug Administration (FDA) is a regulatory agency, which enforces Food, Drug and Cosmetic Act (FDCA), Kefauver-Harris Amendments, Medical Device Amendments and Public Health Service Act (PHS) for rules and conditions to evaluate product safety and efficacy in the United States of America (USA) medical care.^68^ In this current decade, to utilize the safer health care innovations with an involvement to technically advance innovative medicinal therapy, several expedited approval pathways, alternative pathways with exemptions and special situation based decisions have been structured and enacted into legislations for further approval at a short period of time.^69^ Recently FDA guidance expedited program includes Fast Track designation and Breakthrough Therapy designation were created through regenerative medicine advanced therapy (RMAT) designation program by 21st Century Cures Act for the sponsors of regenerative medicine products.^12^

The regulatory criteria for human cells, tissues, and cellular & tissue-based products (HCT/P) issued by the FDA of the US are followed by the Center for Biologics Evaluation and Research (CBER) under section 361 of the Public Health Service Act (PHS) provided in 21 CFR (Code of federal regulations) Parts 1270 and 1271 regulations.^3^ Additionally, for some HCT/Ps not found in the mentioned criteria of 1271.10(a), it could be regulated as drugs, devices or biologics under section 351 of the Public Health Service Act (PHS) and Federal Food, Drug, and Cosmetic Act (FD&C 23Act).^70^ Due to the interdisciplinary landscape, HCT/Ps products with some exemptions such as cryopreserved femoral vein for AV shunt, cultured cells on biomaterial/decellularized scaffolds are regulated as a medical device with tissues or combination products through Center for Devices and Radiological Health (CDRH).^71^ These regulations include HCT/P criteria for minimal manipulation (possibilities of processing method to induce alteration characteristics of the cells or tissue to replace/restore the function), homologous usage (without the combination of other articles which could give rise to new clinical application), its ability of with or without systemic effect having a dependency on cells metabolic activity.^70^ Moreover, this can also regulate the usage of only FDA-registered resource materials for medical application. Further, it also states that manufacturers of HCT/P should register and list the product for approval from the authorities for the license, permission of preclinical, clinical data approval evaluation (Table 6). To facilitate updating, electronic submission is also possible using electronic HCT/P establishment registration (eHCTERS) system with all the submission of requirements.

**Table 6.** Regulations and process details of FDA based Center for Biologics Evaluation and Research (CBER)^72,73^.

FDA approval process	Functions
Investigational new drug (IND) or device exemption (IDE) Process	Request FDA to grant permission to administer drug/medical products to humans
Expanded access to experimental biologics	Request access for the use of non-FDA approved medical products
Biologics license application (BLA) process	Request to obtain a license by a manufacturer or an applicant before marketing after submitting manufacturing methods, pre-clinical and clinical data for evaluating safety, quality and efficacy
Premarket notification (510 k) process	Submission to FDA to demonstrate the efficacy and safety of the product to market it
Premarket approval (PMA) process	Process of FDA to evaluate the safety and efficacy of the approached medical product (mostly Class III medical devices)

FDA is also providing special protocol assessment (SPA) guidance by the Center for Drug Evaluation and Research (CDER) and the Center for Biologics Evaluation and Research (CBER) to help in study designs animal studies and clinical studies and trials. Under section 505(b)(5)(B) of the FD&C Act, under SPA, drug stability or animal efficacy protocols should apply for approval with study designs and statistical analysis. Submission of SPA request is a process that involves (i) Informing FDA of an upcoming request; (ii) Timing of a request for review process up to 45 days which includes documents submission and resubmission of additional required documents; (iii) The request format should contain the cover letter with bold block letters mentioning “**REQUEST FOR SPECIAL PROTOCOL ASSESSMENT**”; (iv) The SPA should submit to the appropriate CDER or CBER division, using standard or electronic submission. FDA will start analyzing the submissions to see whether it is suitable under SPA and communicate decision by mail within 45 days of the review timeline.^74^

#### EU based regulations

In the EU (European Union), classification of tissue engineered products are clearly explained and regulated well and Regulation (EC)No 1394/2007 was designed and amended on December 30, 2008 for Advanced therapy and medicinal products (ATMP including cell therapy, gene therapy and tissue engineered medicinal products) for evaluation of product quality, efficacy and safety.^17,75^ Product description, scientific and technical requirements for improved quality, safety and efficacy are followed through Directive 2001/83/EC, which states that cells are considered to be engineered if it is manipulated including cutting, shaping, centrifugation, sterilization procedures, cryopreservation, lyophilization, etc. Likewise, Directive 2004/23/EC and Directive 2001/20/EC, Regulation (EC) No 726/2004, Directive 2005/28/EC, 2003/94/EC are followed by European Medicines Agency (EMA) for marketing authorization requirements and regulation procedure.^76^ These directives state that the applicants producing product specifications should provide the product details clearly including, name, composition, quantitative & qualitative details, clinical particulars, its interaction with other substance & molecule, precautions steps if any, usage during pregnancy & lactation details, pharmacological, pharmacodynamics & pharmacokinetic properties, pre-clinical safety data, shelf life, storage condition and its procedure, marketing details, etc. In addition, packaging should mention the marketing authorization, manufacture batch number 2004/23/EC, expiry date, method of use, special warnings, etc.^76^ Further, all the clinical data analysis, proper GMP and GLP activities are expected. Particularly, conditional approval has been described by the regulation (EC)N 507/2006 with some difference in the regulatory framework of EU.^17^ EU regulatory implements adaptive approach regulatory pathways mostly for the cellular and tissue-based products. Besides, other pathways are also there such as standard pathway, conditional pathway, adaptive licensing and PRIority MEdicines (PRIME) are framed to regulate products of different categories.^8,12^

#### India based regulations

In India, Drug and Cosmetic Act 1940 and Rules 1945 were enacted to regulate drug approval by Central Drugs Standard Control Organization (CDSCO).^66^ Especially Cell Biology Based Therapeutic Drug Evaluation Committee (CBBTDEC) by CDSCO was formed in 2010 for cell therapy related clinical approval.^77^ Additionally in India, the Indian Council of Medical Research (ICMR) involve in providing guidelines for conducting biomedical and clinical research activities for clinical regulation. Briefly, the Drugs & Cosmetics Act 1940 was legislated by Indian law in concern with the importing, manufacturing and marketing drugs and cosmetics commercially to all over India with assured safety and high quality in their role. In accordance with this act, CDSCO has established Drugs Technical Advisory Board (DTAB) and Drugs Consultative Committee (DCC) with the main objective to maintain and follow common standards in commercializing, clinically practicing the new drug/existing drug for human disease treatment. Tissue engineered products including stem cell-based products are categorized as a drug in evaluating its clinical practice application, regulation and for its clinical practice by CBBTDEC. Importantly, requirements governing the clinical trial approvals, license and permissions are covered under the Schedule Y, Rules 122 A, 122 B, 122 DA, 122 DB, 122 DAC, 122 DD.^66^ Product safety and efficacy is evaluated through the Chemistry, Manufacturing and Control (CMC) data, pre-clinical data and clinical trial data (phase I, phase II, and phase III). Moreover, ICMR had launched ethical guidelines for biomedical research on a human subject in 2000 (revised in 2006 and 2017) and good clinical laboratory practice guidelines (2008) for the conduct of clinical trials in India.^77^ Good clinical practice (GCP) guidelines provide standards for obtaining quality data by designing reproducible clinical work protocol in the research laboratories to obtain the same quality, reliable results, meanwhile saving money, time and complexity involved in experiments.

#### Regulations followed by other countries

Likewise in Japan, Pharmaceuticals and Medical Devices Agency (PMDA) regulates the tissue engineered products to clinical practice through the Safety and Regenerative Medicine Act (RM Act) and Pharmaceuticals, Medical Devices and Other Therapeutic Products Act (PMD Act).^78^ In China, Tissue Engineered Medical Products (TEMP) are regulated through different categories as class III medical device based on the classification mentioned in the Medical Devices Classification Rule (MDCR, CFDA Order No. 15) by China Food and Drug Administration(CFDA).^15^ In China, the regulatory approach is mainly focused on the final product utility in treating the medical need and it does not mainly depend on the raw material and processing method.

A cohort study conducted by Coppens on the approval of GCTs between 2008 and 2017 by US, EU and Japan regulatory concluded that Japan showed higher acceptance of GCT in consideration with uncertainties and safety risks followed by EU and US. In the majority of cases, unmet medical needs are considered for its regulatory approval by the EU and Japan clinical transformation regulatory system. While considering post-marketing characteristics, product safety, quality and risks are analyzed in Japan and EU, but in US safety is the main focus.^8^ However, US and EU have many alternative pathways and regulations to facilitate the approval process relatively faster. Japan follows time limited approval pathways to overcome some of the already existing limitations.^64^

### Regulations for 3D printed/bioprinted TEMPs

3D printed (additive manufactured) products in medical applications are fabricated as whole device/part which could be utilized as prosthesis or an implant or other medical assistance devices.^73^ In recent times, 3D printed prostheses for hip, knee, skull, jaw bone or joint implant, limb prostheses, orthopedic implants, heart valves, etc., are developed and currently under extensive clinical research. These 3D printed prostheses have the potential to create personalized implants of complex structures with precisely controlled material and structural properties within short time when compared to other implant fabrication methods.^2,53,79,80^ Further, additive manufactured metal implants have added advantage in controlling internal porous structures to promote biological fixation with long-term stability. Although 3D-printed implants (including metal implants) are devoid of cellular materials, they are also regulated as medical devices.^81^ However, bioprinted TEMPs with complex cell source and bioink composition for regenerative medicinal application could be regulated either as biologics or drugs/medical devices based on the regulatory authorities.^82^ 3D bioprinted tissues fall between the categories of living materials & technology and hence do not fall directly into the existing categories of regulations. In order to bring this to light, experts have developed a concept called “bio-objects,” which means that all kinds of biotechnologies fall “in-between” the existing categories of living and non-living matter.^83,84^ These “bio-objects” do not fall into the standard regulatory frameworks which are based on the clinically well-established medicinal product categories. Consequently, these products require new regulations/laws for clinical trials and commercialization.

In the US, personalized 3D printed medical devices are regulated under the medical device category or with some custom device exemptions to evaluate their safety and efficacy through pre-market/post-market requirements.^85^ However, generic medical devices are categorized into 16 special domains (based on their usage and risk) and each device is considered among one of the three regulatory classes based on its safety level and efficacy. These medical devices are classified through *de novo* classification—a risk-based classification for the regulations as mentioned in Table 7. Based on these categorical classifications, 510 k regulation is required for class I and II devices while for class III, premarket approval application (PMA) is required for the FDA approval process. All these classification systems are subject to common control requirements of Food, Drug and Cosmetic Act (FDCA) and additional market approval as a design control model from regulatory agencies.^69^

**Table 7.** Classifications of medical devices (TEMPs) based on risk levels.

US—medical device^86^			
Class I**—**General control (with/without exemptions)	Class II**—**General control and specific control (with/without exemptions)		Class III**—**General control with premarket approval
• Low risk For example, breast pump, stethoscope, etc.	• Moderate risk device For example, catheter, bone plate, joint implant, etc.		• Highest risk devices For example, Mechanical heart valve, implantable infusion pump, heart stent, hip prostheses, etc.
EU—medical device^87^			
Class I	Class IIa	Class IIb	Class III
• Low risk • Noninvasive device For example, stethoscopes, electrodes (EEG/ECG), etc.	• Low to medium risk Surgically invasive/noninvasive, active and nonhazardous to patient For example, infusion cannula, surgical swabs, needle for suturing, etc.	• Medium to high risk • Invasive/noninvasive, active devices, implantable • (Has some hazardous effect) For example, catheters, peripheral vascular graft and stent, maxillofacial implants, etc.	• High risk Supports human life in direct connection with health system For example, Neuro-endoscopes, cardiovascular catheter, biological heart valve, etc.

The FDA has provided guidelines for the 3D printed materials under two different considerations such as Design and Manufacturing Considerations (device design/patient match design, software process, material, printing process & control, post-process and validation) and Device Testing Considerations (device description and measurements, material characterization and mechanical testing) to ensure product quality, efficacy and determination of classification for regulations.^88^ Currently, 3D printed medical devices are regulated through premarket notification, New Drug Application (considering as new drug) and Biologics License Application (considering as biologics).^73^ The FDA has so far approved the AXIOM 20 3D printer for manufacturing medical devices and one 3D printed drug named Spritam^®^ tablets for epilepsy treatment.^89^

On the other hand, EU follows legislation such as AIMDD 90/385/EE (Active Implantable Medical Device Directive), MDD 93/42/EEC (Medical Device Directive) and IVDMDD 98/79/EC (In Vitro Diagnostic Medical Device Directive) for 3D printed medical device products. Further, as per the standards mentioned in directive 93/42/EEC, medical devices are classified into four categories such as Class I (Noninvasive devices), Class IIa, Class IIb and Class III ranked from lowest to higher risk (Table 7). Higher rank classes require higher safety assessment levels and such classification levels are decided based on the consideration of patient contact duration, degree of invasiveness and place of the implantation/contact in human body part. These rules have been followed by effectively implementing as a set of 18 rules presented in Table 8. A titanium based (Ti-6Al-4V) 3D printed implant called iFuse-3D is clinically used as structural support for sacroiliac joint to promote bone growth. This implant has received market clearance under conventional medical devices regulations in both US and EU. Post-market evaluation of this implant has assessed patient safety and efficacy of the product similarly to a machined implant. The clinical outcomes had revealed that iFuse-3D reduced the pain related complaints, thereby avoided secondary corrective surgeries. In addition, this implant did not show any unanticipated clinical complaints after treatment and hence is efficient in creating patient-specific implants using 3D printing technology.^90^ Likewise, Ackland et al. fabricated personalized 3D printed prosthetic temporomandibular joint (TMJ) for a 58-year female patient. This prosthetic was modeled using patient musculoskeletal modeling for assessing implant stress and strain, applied load, screw stress and other physiological loadings. Further, the modeled prosthetic was analyzed, fabricated and finally implanted into the patient to study its efficacy. It was observed that a normal jaw opening distance of 40.0 mm was achieved with less pain after 6 months of postoperative surgery. These results demonstrate the effectiveness of personalized 3D printed complex joint replacement prosthetics developed through several modeling and computational analysis.^91^ Hyun Ho Han et al. have fabricated patient-specific, 3D printed biodegradable scaffolds using medical-grade polycaprolactone and implanted them in three patients with complex maxillary defects. This implant is registered as a medical device in South Korea (registration No. 14–1337) and manufactured according to Good Manufacturing Practice provisions (registration No. KTC-ABB-170177). In the follow-up period of 16 months, implanted scaffolds promoted neo-tissue ingrowth which was confirmed by CT images compared between pre and post-surgery images (2711 mm^3^) and Hounsfield unit values (preoperative - −76.269; after 6  and 16 months of implantation - +63.7825 and +73.0488).^92^

**Table 8.** EU directives and classification rules for medical devices as per annexure IX of directive 93/42/EEC^87^.

Category	Rules	Indications	Medical device class category
Non-Invasive	Rule 1	Do not touch patient/contact only skin	Class I
	Rule 2	Channeling or storing for eventual administration	Class I
			Class IIa (if it is used with blood or used along with active device of class IIa and higher classes)
	Rule 3	Modify biological or chemical composition of blood, body fluid, etc. for infusion	Class IIb
			Class IIa (only filtration, centrifugation, or gas exchange)
	Rule 4	In contact with injured skin	Class I
			Class IIa (intended to manage micro environment of skin)
			Class IIb (intended for wounds which breach dermis and needs secondary intent for healing)
Invasive	Rule 5	Invasive in body orifice or stoma (not surgically invasive)	Class I (transient use, sort term use only in oral cavity/ear canal/nasal cavity)
			Class IIa (short term use, connected to active medical device in class IIa and higher device, long term use in oral cavity/ear canal/nasal cavity)
			Class IIb (long term use)
	Rule 6	Surgically invasive Transient use	Class I (reusable surgical instrument)
			Class IIb (supply energy/ionizing radiation, biological effect mainly/wholly absorbed, intended to administer medicine in a potentially hazardous manner)
			Class III (to monitor/control/diagnose/correct defect in heart or central nervous system (CNS) by direct contact
	Rule 7	Surgically invasive Short term use	Class IIb (supply energy/ionizing radiation, undergo chemical change in body or administer medicines)
			Class III (to monitor/control/diagnose/correct defect of heart or central nervous system (CNS) by direct contact)
	Rule 8	Surgically invasive Long term use and implantable device	Class IIa (placed in teeth)
			Class III (in direct contact with heart or CNS, biological effect or mainly absorbed, undergo chemical change in body or administer medicine, breast implant, hip, knee and shoulder joint replacement
Active devices	Rule 9	Active therapeutic devices intended to administer or exchange energy	Class IIa
			Class IIb (if it is hazardous way, intended to control/monitor/influence directly the performance of class IIb active therapeutic devices)
	Rule 10	Active device for diagnosis, is intended to supply energy, to image *in vivo* distribution of radiopharmaceuticals or for direct diagnosis or monitoring of vital physiological process	Class IIa
			Class IIb (intended to monitor vital physiological parameters where variations could result in immediate danger, all devices emitting ionizing radiations and intended for diagnostic and therapeutic interventional radiology
	Rule 11	Active devices to administer or remove medicines and other substances to or from the body	Class IIa
			Class IIb (if it is in a potentially hazardous way)
	Rule 12	All other active devices	Class I
Special rules	Rule 13	Device incorporating integral medicinal substance liable to act in ancillary way on human body	Class III
	Rule 14	Device used for contraception or prevention of sexually transmitted diseases	Class IIb
			Class III (if implantable or long-term invasive)
	Rule 15	Specifically, to be used for disinfecting medical devices	Class IIa
			Class IIb (specifically to be used for disinfecting invasive devices, disinfecting, cleaning, rinsing or hydrating contact lenses)
	Rule 16	Devices intended for recording of X-ray diagnostic images	Class IIa
	Rule 17	Device utilizing non-viable animal tissues or derivatives (not devices in contact with intact skin)	Class III
	Rule 18	Blood bags	Class IIb

## Universal standards for tissue engineered products

Standards for TEMPs are established to ensure product characteristics and all associated analytical procedures to ensure common quality results worldwide with improved repeatability and reliability of the data. The International Organization of Standards (ISO) and the American Society for Testing and Materials (ASTM) International are the recognized organizations for presenting standardization of biomaterials and medical devices.^93^ ASTM International is an organization that provides resources, technical expertise to develop universal standards and guidelines for a wide range of developing fields. It comprises several committees and subcommittees with respect to specific technical areas to frame the standards and guidelines including material characteristics, test methods, system, etc. Specifically for tissue engineered medical products, the F04 committee on “Medical and Surgical Materials and Devices” with other subcommittees named Division IV “Tissue-Engineered Medical Products (TEMPs)” was established in 1997 and the up to date standards published by these committees (Table 9).^94^ Further, ASTM has established the F42 committee on “Additive Manufacturing Technologies” in 2009 with eight technical committees to formulate standards for 3D printed medical products.^89^

**Table 9.** List of published ASTM standards framed by committee F04 and sub-committee IV for TEMPs.^95^

ASTM subcommittee	Category	Published standards (ASTM)	
F04.41	Classification and terminology for TEMPs	ASTM F2211-13	Standard specification for general classification for tissue engineered medical products
		ASTM F2312-11(2020)	Standard terminology relating to tissue engineered medical products
		F3163-16	Standard guide for classification of cellular and/or tissue-based products (CTPs) for skin wounds
F04.42	Biomaterials and biomolecules for TEMPs	F2027-08(TG13 WK9442)	Standard guide for characterization and testing of raw or starting biomaterials for tissue engineered medical products
		F2027-16	Standard guide for characterization and testing of raw or starting materials for tissue-engineered medical products
		F2064-17	Standard guide for characterization and testing of alginates as starting materials intended for use in biomedical and tissue engineered medical product applications
		F2103-18	Standard guide for characterization and testing of chitosan salts as starting materials intended for use in biomedical and tissue-engineered medical product applications
		F2131-02(2012) & WK67352	Standard test method for in vitro biological activity of recombinant human bone morphogenetic protein-2 (rhBMP-2) Using the W-20 mouse stromal cell line
		F2150-19	Standard guide for characterization and testing of biomaterial scaffolds used in regenerative medicine and tissue-engineered medical products
		F2212-20, WK68915 and WK70847	Standard guide for characterization of Type I collagen as starting material for surgical implants and substrates for tissue engineered medical products (TEMPs)
		F2259-10(2012)e1	Standard test method for determining the chemical composition and sequence in alginate by proton nuclear magnetic resonance (1H NMR) spectroscopy
		F2260-18	Standard test method for determining degree of deacetylation in chitosan salts by proton nuclear magnetic resonance (1H NMR) spectroscopy
		F2347-15	Standard guide for characterization and testing of hyaluronan as starting materials intended for use in biomedical and tissue engineered medical product applications
		F2450-18	Standard guide for assessing microstructure of polymeric scaffolds for use in tissue-engineered medical products
		F2602-18	Standard test method for determining the molar mass of chitosan and chitosan salts by size exclusion chromatography with multi-angle light scattering detection (SEC-MALS)
		F2603-06(2020)	Standard guide for interpreting images of polymeric tissue scaffolds
		F2605-16	Standard test method for determining the molar mass of sodium alginate by size exclusion chromatography with multi-angle light scattering detection (SEC-MALS)
		F2791-15	Standard guide for assessment of surface texture of non-porous biomaterials in two dimensions
		F2952-14	Standard guide for determining the mean darcy permeability coefficient for a porous tissue scaffold
		F3089-14	Standard guide for characterization and standardization of polymerizable collagen-based products and associated collagen-cell interactions
		F3142-16	Standard guide for evaluation of in vitro release of biomolecules from biomaterials scaffolds for TEMPs
		F3259-17	Standard guide for micro-computed tomography of tissue engineered scaffolds
		F3354-19	Standard guide for evaluating extracellular matrix decellularization processes
		F3510-21	Standard guide for characterizing fiber-based constructs for tissue-engineered medical products
F04.43	Cells and tissue engineered constructs for TEMPs	F2149-16	Standard test method for automated analyses of cells—the electrical sensing zone method of enumerating and sizing single cell suspensions. document to be reviewed for possible updating of data evaluation section
		F2315-18	Standard guide for immobilization or encapsulation of living cells or tissue in alginate gels
		F2664-19e1	Standard guide for assessing the attachment of cells to biomaterial surfaces by physical methods
		F2944-20	Standard practice for automated colony forming unit (CFU) assays—image acquisition and analysis method for enumerating and characterizing cells and colonies in culture
		F2997-13	Standard practice for quantification of calcium deposits in osteogenic culture of progenitor cells using fluorescent image analysis
		F3088-14	Standard test method for use of a centrifugation method to quantify/study cell-material adhesive interactions
		F3106-14	Standard guide for in vitro osteoblast differentiation assays
		F3206-17	Standard guide for assessing medical device cytocompatibility with delivered cellular therapies
		F3369-19e1	Standard guide for assessing the skeletal myoblast phenotype
		F3209-16	Standard guide for autologous platelet-rich plasma for use in tissue engineering and cell therapy
		F2739-19	Standard guide for quantitating cell viability within biomaterial scaffolds
F04.44	Assessments for TEMPs	F2451-05	Standard guide for the assessment of implantable devices intended to repair or regenerate articular cartilage
		F2529-13	Standard guide for in vivo evaluation of osteoinductive potential for materials containing demineralized bone (DBM)
		F2884-12	Standard guide for pre-clinical *in vivo* evaluation of spinal fusion
		F3207-17	Standard guide for *in vivo* evaluation of rabbit lumbar intertransverse process spinal fusion model
		F3223-17	Standard guide for characterization and assessment of tissue engineered medical products (TEMPs) for knee meniscus surgical repair and/or reconstruction
		F3224-17	Standard test method for evaluating growth of engineered cartilage tissue using magnetic resonance imaging
		F3225-17	Standard guide for characterization and assessment of vascular graft tissue engineered medical products (TEMPs)
		F3368-19	Standard guide for cell potency assays for cell therapy and tissue engineered products
		F2721-09(2014)	Standard guide for pre-clinical *in vivo* evaluation of critical size segmental bone defects
F04.45	Safety	WK70143 (proposed)	Sampling methods of tissue engineered medical products (TEMPs) for sterility assurance

Similarly, ISO comprises a Technical Committee (TC) and Sub Committee (SC) to provide standards and guidelines for different fields. In particular, for fields of tissue engineering and regenerative medicine, technical committee 150 “Implants for surgery” with subcommittee SC7 “Tissue-engineered medical products,” which was established in 2001 covers TEMPs based on published standards (International organization of standards/Technical committee/Subcommittee ISO/TC 150/SC7) (Table 10). Other technical committees such as TC194 (Biological and clinical evaluation of medical devices), TC210 (Quality management and corresponding general aspects for medical devices), TC212 (Clinical laboratory testing and in vitro diagnostic test systems), TC261 (Additive manufacturing) and TC266 (Biomimetics) are established with several standards which are widely utilized for health care applications.^124^ Periodically, each committee could establish the new standards or revised version of published standards for evaluation. For example, TC150 has published 165 standards and TC 194 has published 32 ISO standards in 2020. An informative list of ASTM and ISO standards followed by world regulatory authorities for commercializing medical products are tabulated (Table 11). These standards and updated protocols could help to establish a uniform approach and assessment procedure for validating medical products including tissue engineering products while ensuring high quality and more safety to humans.^115^ This is imperative because cellular and acellular synthetic/natural constructs need specific standards for characterization and in vitro, *in vivo* and clinical trial analyses with respect to the raw material used.^114^

**Table 10.** List of published ISO standards of technical committee 194 and 150 for the regulation of medical device and tissue engineered products (TEMPs).

TC/SC	Published standards	
TC 194/SC1 Tissue product safety (“ISO—ISO/TC 194/SC 1—tissue product safety)^96^	ISO 13022:2012	Medical products containing viable human cells—application of risk management and requirements for processing practices
	ISO 22442-1:2015	Medical devices utilizing animal tissues and their derivatives—part 1: application of risk management
	ISO 22442-2:2015	Medical devices utilizing animal tissues and their derivatives—part 2: controls on sourcing, collection and handling
	ISO 22442-3:2007	Medical devices utilizing animal tissues and their derivatives—part 3: validation of the elimination and/or inactivation of viruses and transmissible spongiform encephalopathy (TSE) agents
TC 150/SC7 Tissue engineered medical products (TEMP) (“ISO—ISO/TC 150/SC 7—tissue-engineered medical products)^97^	ISO 13019:2018	Tissue-engineered medical products—quantification of sulfated glycosaminoglycans (sGAG) for evaluation of chondrogenesis
	ISO/TR 16379:2014	Tissue-engineered medical products—evaluation of anisotropic structure of articular cartilage using DT (diffusion tensor)—MR Imaging
	ISO 19090:2018	Tissue-engineered medical products—bioactive ceramics—method to measure cell migration in porous materials
	ISO/CD TS 21560.2020	General requirements of TEMPs
	ISO 7198:2016	Cardiovascular implants and extracorporeal systems—vascular prosthesis—tubular vascular grafts and vascular patches
TC168 prosthetics and orthotics (“ISO—ISO/TC 168—prosthetics and orthotics)^98^	ISO 13405-1:2015	Prosthetics and orthotics—classification and description of prosthetic components—part 1: Classification of prosthetic components
	ISO 13405-2:2015	Prosthetics and orthotics—classification and description of prosthetic components—part 2: Description of lower limb prosthetic components
	ISO 13405-3:2015	Prosthetics and orthotics—classification and description of prosthetic components—part 3: Description of upper limb prosthetic components
	ISO 21065:2017	Prosthetics and orthotics—terms relating to the treatment and rehabilitation of persons having a lower limb amputation
	ISO 8549-1:2020	Prosthetics and orthotics—vocabulary—part 1: general terms for external limb prostheses and external orthoses
	ISO 8549-2:2020	Prosthetics and orthotics—vocabulary—part 2: terms relating to external limb prostheses and wearers of these prostheses
	ISO 8548-1:1989	Prosthetics and orthotics—limb deficiencies—part 1: method of describing limb deficiencies present at birth
	ISO 8548-2:2020	Prosthetics and orthotics—limb deficiencies—part 2: Method of describing lower limb amputation stumps
	ISO 8548-3:1993	Prosthetics and orthotics—limb deficiencies—part 3: Method of describing upper limb amputation stumps
	ISO 8548-4:1998	Prosthetics and orthotics—limb deficiencies—part 4: Description of causal conditions leading to amputation
	ISO 8548-5:2003	Prosthetics and orthotics—limb deficiencies—part 5: Description of the clinical condition of the person who has had an amputation
	ISO 29783-1:2008	Prosthetics and orthotics—vocabulary—part 1: normal gait
	ISO 29783-2:2015	Prosthetics and orthotics—vocabulary—part 2: Prosthetic gait
	ISO 8549-3:2020	Prosthetics and orthotics—vocabulary—part 3: terms relating to orthoses
	ISO 8549-4:2020	Prosthetics and orthotics—vocabulary—part 4: terms relating to limb amputation

**Table 11.** Established ISO & ASTM standards applied for global tissue engineered product regulation from basic pre-clinical research to clinical trial research.

Published ISO standards		Ref
ISO 14155:2020	Clinical investigation of medical devices for human subjects—Good clinical practice	Bosiers et al.^99^
ISO 22442-1:2020	Medical devices utilizing animal tissues and their derivatives—part 1: Application of risk management	ISO^100^
ISO 22442-2:2020	Medical devices utilizing animal tissues and their derivatives—part 2: Controls on sourcing, collection and handling	ISO^101^
ISO 10993-1:2018	Biological evaluation of medical devices—part 1: evaluation and testing within a risk management process	Parente^102^
ISO 10993-2:2006	Biological evaluation of medical devices—part 2: animal welfare requirements	Singh et al.^103^
ISO 10993-3:2014	Biological evaluation of medical devices—part 3: tests for genotoxicity, carcinogenicity and reproductive toxicity	De Moura and Van Houten^104^
ISO 10993-7:2008/AMD 1:2019	Biological evaluation of medical devices—part 7: ethylene oxide sterilization residuals—amendment 1: applicability of allowable limits for neonates and infants	Gimeno et al.^105^
ISO 10993-9:2019	Biological evaluation of medical devices—part 9: framework for identification and quantification of potential degradation products	Reeve and Baldrick^106^
ISO 10993-12:2012	Biological evaluation of medical devices—part 12: sample preparation and reference materials	Coleman et al.^107^
ISO 10993-13:2010	Biological evaluation of medical devices—part 13: identification and quantification of degradation products from polymeric medical devices	Deliversky et al.^108^
ISO 10993-14:2001	Biological evaluation of medical devices—part 14: identification and quantification of degradation products from ceramics	Deliversky et al.^108^
ISO 10993-15:2019	Biological evaluation of medical devices—part 15: identification and quantification of degradation products from metals and alloys	Horicsányi et al.^109^
ISO 10993-18:2020	Biological evaluation of medical devices—part 18: chemical characterization of medical device materials within a risk management process	Jóźwicka et al.^110^
ISO 22196:2011	Measurement of antibacterial activity on plastics and other non-porous surfaces	Qureshi et al.^111^
ISO13314:2011	Mechanical testing of metals—ductility testing—compression test for porous and cellular metals	Zaharin et al.^112^
ISO 13485:2016	Medical devices—quality management systems—requirements for regulatory purposes	Bhat et al.^89^
ISO 7198:1998	Specific requirement for tubular prostheses for vascular functions	Aussel et al.^113^
ISO 21536:2007/AMD 1:2014	Non-active surgical implants—joint replacement implants—specific requirements for knee-joint replacement implants—amendment 1	Marchiori et al.^114^
ISO 14630:2012	Non-active surgical implants—general requirements	
ISO 5840-1:2015	Cardiovascular implants—cardiac valve prostheses—part 1: general requirements	Zhang et al.^115^
ISO 9001:2015	Quality management systems—requirements	Bt Hj Idrus et al.^16^
ISO/IEC 17025:2017	General requirements for the competence of testing and calibration laboratories	
ISO 13019:2018	Tissue-engineered medical products—quantification of sulfated glycosaminoglycans (sGAG) for evaluation of chondrogenesis	ISO^116^
ISO/TR 16379:2014	Tissue-engineered medical products—evaluation of anisotropic structure of articular cartilage using DT (Diffusion Tensor)-MR Imaging	Tensor^117^
ISO 19090:2018	Tissue-engineered medical products—bioactive ceramics—method to measure cell migration in porous materials	ISO^118^
ISO/TS 21560:2020	General requirements of tissue-engineered medical products	ISO^119^
Active ASTM standard		
ASTM F2150-19	Standard guide for characterization and testing of biomaterial scaffolds used in tissue-engineered medical products	Tesk^93^
ASTM F2211-13	Standard classification for tissue engineered medical products (TEMPs)	Tesk^93^
ASTM F2312-11	Standard terminology relating to tissue engineered medical products	Lee et al.^72^
ASTM F2739-19	Standard guide for quantitating cell viability within biomaterial scaffolds	Oyama et al.^120^
ASTM F2315-18	Standard guide for immobilization or encapsulation of living cells or tissue in alginate gels	Lee et al.^72^
ASTM F3142-16	Standard guide for evaluation of in vitro release of biomolecules from biomaterials scaffolds for TEMPs	Garcia et al.^121^
ASTM F1635-16	Standard test method for in vitro degradation testing of hydrolytically degradable polymer resins and fabricated forms for surgical implants	Whitfield-Gabrieli and Nieto-Castanon^122^
ASTM F2027-1.6	Standard guide for characterization and testing of raw or starting biomaterials for tissue-engineered medical products	Amato^123^
ASTM F2211-13	Standard classification for tissue engineered medical products (TEMPs).	Tesk^93^
ASTM F2450-18	Standard guide for assessing microstructure of polymeric scaffolds for use in tissue engineered medical products	Garcia et al.^121^
ASTM F2603-06(2012)	Standard guide for interpreting images of polymeric tissue scaffolds	Amato^123^

TC 261 published ISO/ASTM 52910:2018 standard “Additive manufacturing—Design—Requirements, guidelines and recommendations,” which describes the design consideration that needs to be taken while manufacturing different types of products or components through the additive manufacturing approach. This standard will provide necessary guidance to the engineers and students to carry out the basic design of additive manufactured products for clinical translation. These design guidelines facilitate the manufacturers to decide on optimal cost, quality and delivery time. Further, this could also be applied to identify the potential of additive manufacturing techniques by assessing the choice of material, its availability, build volume and print volume comparison.^125^

ISO in liaison with the governmental, non-governmental and international organizations have also been facilitated to further raise the standards with more expertise in the field. For instance, ISO 261 and ASTM F47 have jointly framed general standards (framing additive manufacturing requirements, guidelines, safety, definition and concepts) and specific/broad category of material based standards (framing material, process or application details) to continuously improve worldwide standards and quality of additive manufactured products without any confusions^126^ This also enables the development of new inventions in all fields related to additive manufacturing spanning from the industrial sector to medicine. ISO TC 261 has a group of field expertise called a working group such as WG 1, WG 2, WG 3, WG 4, and WG 6 for the development of standards for manufacturing additive manufactured products. Similarly, several joint groups have been established with different titles such as ISO/TC 261/JG 57 “Joint ISO/TC 261-ASTM F 42 Group: Process-specific design guidelines and standards”, ISO/TC 261/JG 54 “Joint ISO/TC 261-ASTM F 42 Group: Fundamentals of Design”, ISO/TC 261/JG 59 “Joint ISO/TC 261-ASTM F 42 Group: NDT for Additive Manufacturing part”, ISO/TC 261/JG 60 “Joint ISO/TC 261-ASTM F 42 Group: Additive manufacturing—Non-destructive testing and evaluation—Standard guideline for intentionally seeding flaws in parts”, etc.^127^ However, all these established standards of ISO are maintained as a catalog where the standards are classified into different ICS (International classification of standards) with specific description level (field, group, subgroup) with codes for better understanding and followed as required.

## Clinical research guidelines

International Organizations such as the World Health Organization, Council for International Organizations of Medical Sciences (CIOMS), US Department of Health and Human Services, National Institute of Health, Council of Europe and Nuffield Council on Ethics are working to provide guidance for the research on the health care field on ethics, safety and quality medical therapy/products development.^128^ The main objective is to ensure that guidelines should be clear, complete and should take into consideration of all regulatory aspects of the country. To ensure human health with safer medicinal products, several guidelines are being published in collaboration with a wide variety of technical experts of the field to provide all the detailed procedures and requirements with ethical concerns relevant to the particular topic.^16^ Additionally, an International standard for conducting clinical trials named “Good Clinical Practice” was issued by International Conference on Harmonization (ICH).^129^ Furthermore, clinical trials should be conducted under the ethical principles in the Declaration of Helsinki, earlier risk and beneficial assessment, rights, safety and well-being of participants analysis, planned data storage and with approved protocols. All the published, new and revised versions of the guidelines for tissue engineered product regulation of the US, India and European Union are highlighted in Table 12 with a brief description of the guidelines and their scope. The objectives of these regulations are based on providing maximum benefits with minimum risk and hence EC (Ethical Committee), researchers and other stakeholders are always dependent on benefit-risk assessments.^129^ These guidelines helps researchers, industrial manufacturers, marketing authorization members and other involved members about the regulatory need, approach and analysis. Importantly, these guidelines have to be regularly revised, modified and improved envisaging tissue engineered product commercialization in the future.

**Table 12.** Compilation of the published guidelines applicable to US, India and European Union for tissue-based product regulations.

	Year	Guidance/published guidelines	Scope
U.S (FDA) (U.S. Food and Drug Administration (FDA))^132^	2020	Regulatory considerations for human cells, tissues, and cellular and tissue-based products: minimal manipulation and homologous use	For better understanding of terms minimal manipulation and homologous use criteria for tissue engineered product to regulate under 21 CFR Part 1271 and specifically the 21 CFR 1271.10(a)(1) criterion of minimal manipulation and the 21 CFR 1271.10(a)(2) criterion of homologous use
	2019	Enrichment strategies for clinical trials to support approval of human drugs and biological products	Exploring the strategies of patient selection to improve drug potency by decreasing the clinical group variations, improving group prognostics and prediction for the drug specific treatment
	2017	Deviation reporting for human cells, tissues, and cellular and tissue-based products regulated solely under section 361 of the public health service act and 21 CFR part 1271	With respect to the technical innovation and development of tissue-based products this guideline describes the HCT/P deviations clearly for its regulation under 361 of the public health service act and 21 CFR part 1271
	2016	Investigating and reporting adverse reactions related to human cells, tissues, and cellular and tissue-based products (HCT/Ps) regulated solely under section 361 of the public health service act and 21 CFR part 1271	This provides the supplement guidance to the “guidance for industry: current good tissue practice (CGTP) and additional requirements for manufacturers of human cells, tissues, and cellular and tissue-based products (HCT/Ps)” (December 2011) for reporting adverse reactions on the study
	2011	Current good tissue practice (CGTP) and additional requirements for manufacturers of human cells, tissues, and cellular and tissue-based products (HCT/Ps)	For manufacturer’s better understanding of good clinical practice requirements (facilities, environment control, equipment and reagent source criteria, processing and process control, storage, package, shipment, labeling, donor screening testing and eligibility), registration and listing requirements for HCT/Ps regulation
	2008	Certain human cells, tissues, and cellular and tissue-based products (HCT/Ps) recovered from donors who were tested for communicable diseases using pooled specimens or diagnostic tests	Donor eligibility considerations and requirements (screening and testing) to avoid or reduce the communicable disease transmission
	2007	Regulation of human cells, tissues, and cellular and tissue-based products (HCT/Ps) - small entity compliance guide	Provide details for the HCT/Ps product to register (registration form FDA 3356) and list with center for biologics evaluation and research (CBER) with regular updates and complete guidance for its safety evaluation
	2007	Eligibility determination for donors of human cells, tissues, and cellular and tissue-based products	Represent the establishment of donor safety with free of risk factors for the better availability of tissue-based products in medical care applications
	2006	Compliance with 21 CFR Part 1271.150(c)(1)—manufacturing arrangements	Provide requirements for manufacturing if there is another establishment to undertake any manufacture steps to ensure whether good tissue practice (CGTP) requirements are satisfied
	2002	Validation of procedures for processing of human tissues intended for transplantation	In order to avoid infection of any communicable diseases through HCT/Ps it is necessary to prepare all necessary procedures written with complete validation to avoid infections to patient
	1997	Screening and Testing of Donors of Human Tissue Intended for Transplantation	Provide general requirements for screening and testing procedures for the individual involved in whole procedures to ensure complete diagnosis of heath to assure absence of any diseases
India (ICMR) (Indian Council of Medical research (ICMR))^133^	2019	National guidelines for gene therapy product development and clinical trials	States the development of effective and safe GTP with controlled product characterization, quality assessment, preclinical evaluation, clinical study and long-term patient analysis to ensure proper medicinal application
	2017	National ethical guidelines for biomedical research involving children	Detail description about the ethical issue need to take care for biomedical research on children since there is wide variation in metabolic activity, disease, pharmacokinetics, and adverse effect to study the effect of treatment is safer for children
	2017	National ethical guidelines for biomedical and health research involving human participants	It’s a latest version of guideline with incorporation of Indian cultural, social behavioral, and natural environment areas into biomedical research field involving human studies for better medical application
	2017	National guidelines for stem cell research	Guidelines providing procedures, regulatory pathways, research works on human stem cells with proper ethical and scientific considerations
	2008	Guidelines for good clinical laboratory practices (GCLP)	Provide principles and procedures for the clinical trial laboratories to produce quality results with assured human safety
	2006	Ethical guidelines for biomedical research on human participants	Earlier versions of ethical guidance for clinical research in India
EU (EMA)^134^	2019	Guidelines on quality, non-clinical and clinical requirements for investigational advanced therapy medicinal products in clinical trials	Provide data requirement and other need for manufacturing, development, quality control for clinical and non-clinical studies of ATMP
	2018	Guideline on the quality, non-clinical and clinical aspects of gene therapy medicinal products	Provide specific requirements for Marketing Authorization Application (MAA) and regulation of GTPs with respect to quality, nonclinical and clinical aspects
	2017	Guideline on strategies to identify and mitigate risks for first-in-human and early clinical trials with investigational medicinal products	It’s a first revision published with first-in-human (FIH) and phase clinical trial (CT) study with their EU regulations to study safety, tolerability, pharmacokinetic and pharmacodynamics of the investigational medicinal product
	2016	Guideline on potency testing of cell-based immunotherapy medicinal products for the treatment of cancer	It provides guidance for the specific requirement of assays and procedures for the cell-based immune therapy products for quality control and manufacture
	2014	Reflection paper on clinical aspects related to tissue engineered products	Guidance for the clinical testing of tissue engineered products under regulation (EC) No 1394/2007
	2013	Guideline on the risk-based approach according to annex I, part IV of directive 2001/83/EC applied to Advanced therapy medicinal products	Describes the importance and assessment of risk-based approaches for the regulation of advanced therapy medicinal products (ATMP) under directive 2001/83/EC
	2011	Reflection paper on stem cell-based medicinal products	Provide requirements of stem cell-based products marketing authorization application (MAA) and it should be considered along with “guideline on human cell-based medicinal products”
	2008	Guidelines on safety and efficacy follow-up and risk management of advanced therapy medicinal products	Provide characteristics of ATMPs, describes requirements and procedures for the risk-based analysis, safety and efficacy study, clinical aspects and marketing of ATMPs
	2008	Guidelines on human cell-based medicinal products	Provide the guidelines for the quality control, manufacturing approaches and safe human cell-based medicinal products development

On a similar note, ICMR in India formulated the first guidelines, “Ethical guidelines for biomedical research on human participants” in 2006 to conduct human trial research with proper safety measures and effective utilization of study for further applications. It states to conduct human research with four basic principles that include person’s beneficence, non-maleficence, respect and justice to protect participant dignity, safety, rights and wellbeing. Despite various ambiguity and lack of clarity in 2006 guidelines, the following are some technical factors to be considered while revising in future:^130^

i. Possibility of allowing clinical trial for drugs, which are under approval stage by Drug controlled general of India (DCGI)
ii. Not stated regarding the prior agreement for patients benefits after the study and possibility of patient withdrawal should be clear based on the study
iii. Permission for conducting epidemiological studies on school children of age less than 18 years should be clear with parent’s decision
iv. Scientific review and approval of the proposal by the Institutional Ethical Committee (IEC) should be established

This guideline was recently revised in 2017 and renamed as “National Ethical Guidelines for Biomedical and Health Research Involving Human Participants” that covers the ethical lagging for the already existing and new research fields with several updates including a risk-based approach for product categorization and with main considerations of resource availability.^131^ Further, the newly included sections such as Responsible Conduct of Research (RCR), public health research, socio-behavioral research, and research during humanitarian disasters and emergencies provide clear guidelines to all the end-users. This revised version helps to better understand the ethics, importance of research/product quality and personal safety for biomedical and human research.^131^ It has been made mandatory that all clinical trials conducted in India should be registered in Clinical Trials Registry–India (CTRI) (launched in 2007). Likewise, the newly established, updated and available guidelines needed for the medicinal tissue-based regulation for European Union and US are tabulated (Table 12).

## Current status toward clinical translation of engineered tissue constructs

In a recent report, it was observed that upon the interest of Tissue Engineered (TE) product research & development, there have been 66 on-going or approved TE clinical trials (biomaterial/stem cell) between 2011 and 2018 in the United States, indicating a trajectory of TE product growth toward clinical testing.^135^ The global tissue engineering market report 2019 had concluded that North America (43%) and Europe (35%) holds the largest marketing shares of TE products and further reported that the worldwide tissue engineering market reached 14,000 million USD in 2018 and it is expected to reach 55,200 Million USD by 2025.^136^ On the other hand, bioprinted tissues have major obstacles toward clinical translation due to the degradation profile of the construct, immunogenicity of the bioinks, shape fidelity, durability and difficulty in the incorporation of vascularity and less affordability for the targeted applications.^137,138^ However, 3D bioprinted tissue constructs have several advantages and are gaining momentum toward effective ways to overcome these existing challenges. As an example, the average treatment cost of a single burn patient would be approximately around $88,000, whereas large-scale production of 3D bioprinted skin might cost less than this threshold.^139^ Another issue with the clinical practice of 3D bioprinted engineered tissues would be the timeline for developing matured tissues or organs with respect to patients need. Specifically, developing simple tissues such as tubular constructs (blood vessels, heart valves) and skin requires a shorter time, whereas complex tissues or organs such as bladder, liver, kidneys, etc., may require longer time owing to high cell density, granularity, vascularization and additional time for tissue maturation in vitro.^140^ Yet several manufacturers are utilizing this technique for other applications such as cosmetics, tumor models, drug screening and discovery. To date, there is no single bioprinted FDA-approved product for clinical use that is commercially available in the market. Organovo, a 3D bioprinting company, has successfully bioprinted a liver tissue patch and implanted it in a mice model. This liver patch showed better engraftment, fluid retention and other liver-specific functionality for only up to 35 days. As a result, the manufacturer has committed to continue testing the patch further on large animals before testing on humans.^141^ Patient-specific tooth constructs (8 mm × 8 mm × 20 mm) with human dental pulp stem cell (hDPSCs) were printed by Jonghyeuk Han et al. using PCL, gelatin, hyaluronic acid, glycerol and fibrinogen. 5 mg/mL fibrinogen was used to mimic the pulp portion of teeth and 20 mg/mL of fibrinogen was used to print dentin in the PCL support. hDPSCs cultured in high concentrated fibrinogen exhibited odontogenic differentiation by expressing DMP1 and DSPP markers with higher mineralization. In contrast, low concentrated fibrinogen located in the pulp region maintained cells in the undifferentiated state.^142^ Similarly, autologous cells based 3D printed cartilage constructs were developed by Hee-Gyeong Yi et al. for the augmentative rhinoplasty application using alginate and cartilage-derived ECM bioinks. 3D models were designed by scanning the patient face using FaceGen and further processing using Instep software. The resultant digital file was used to fabricate implants of 38.2 mm × 7.3 mm × 6.1 mm (L × W × H) size and the human adipose stem cell-laden (hASC) bioink was injected. The constructs containing cartilage-decellularized ECM showed higher expression of chondrocyte specific genes such as *SOX9, ACAN*, and *COL21A* and GAG at day 14 and 28 days compared to constructs with alginate.^143^ These studies showed the efficacy of 3D printing and cell therapies in fabricating patient-specific models to treat patients with cartilage defects, pancreatic, heart problems and other major therapies in the future. However, more clinical trials need to do to improve the patient safety and efficiency of the product. Nonetheless, the engineered or bioprinted tissues have various ethical scrutiny while translating from bench/pre-clinical side to bedside, which needs to be addressed in the early stages of research thereby thwarting lavish expense of time and money.

### Major problems in clinical transformation of engineered and bioprinted tissues

Worldwide regulatory considerations are not standardized with a common approach for the TEMPs category and hence there is a need for amendments in current standards and regulations. The technical enforcement of regulatory unions on framing the guidelines, reviewing and decision making may differ across the world based on the regulatory framework considered for its assessment.^144^ However, revisions in legislations and adaption of standards to the current technical developments are still lagging in many cases, thus making it a challenge for clinical translation of TEMPs.^8^ This is because the regulatory bodies take a huge time to comprehend the evolving transformations of new technologies and develop appropriate regulations/laws. Further, the experts of these bodies may not be able to make regulations by anticipating future products and technologies. However, these regulatory bodies may be advocated to form a multi-disciplinary research team that includes scientists, legal experts and social scientists. This team could be a part of the regulatory from the start of the development of a new technology and to form suitable regulations. This idea is developed as RRI: Responsible Research and Innovation which advocates this type of multi-disciplinary integration from the inception of new products development.^82^

As an alternative to overcome these challenges, most regulatory bodies are involved in an adaptive approach for its approval of cellular therapy products, gene therapy products, tissue engineered products and additive manufactured medical devices. TEMPs are complex and non-reliable products due to the incorporation of natural biomaterials and cells, which cause different host reactions to the product in a population. Thus, from a product point of view, scientific uncertainties of TEMPs cause a massive challenge and executional difficulties for regulatory authorities to approve medicinal therapies and products. In addition, most of the clinical trials have ended up with minimal efficiency and/or inducing high risk in patients’ health, thus it gets automatically rejected during the approval process. Nevertheless, these limitations make it difficult to formulate common guidelines for characterizing the material/product and it heavily affects the commercialization of high-risk category products to be legally approved for clinical practice all over the world.^15^ Though suitable TEMPs are designed and studied well, delay in their market approval is due to the many different procedures and licensing steps involved in the regulations. It includes pre-clinical assessment studies (animal model, efficacy study, toxicology study), clinical trial design (experimental group and endpoint design, GCP and GMP maintenance), clinical outcome and approval decision after risk-based quality and efficacy assessments. However, less availability of preclinical results adds to the challenges on approval of engineered tissue under the high-risk categories. In these perspectives, the major challenges faced by researchers, sponsors and other non-medical regime partners are mainly due to stringent regulations, poor knowledge in the requirements to conduct clinical trials, inadequate laboratory practice guidelines and lack of funds that eventually make the clinical translation of tissue-based products more difficult. Furthermore, the unavailability of prior relevant standards also limits most of the clinical trial approval of TEMPs.

A pilot study conducted by Davies et al. in the United Kingdom along with cardiology, neurology, ophthalmology, orthopedic surgery, plastic and reconstructive surgery clinical specialists, have identified the major barrier for clinical transformation as efficacy and cost-effectiveness in addition to other observations including safety, regulation, methodology, visibility and patient characteristics for cell-based therapy clinical treatment. Moreover, it was also noted that inconsiderate processing techniques involved in manufacturing reduce the possibilities of approval through regulatory pathways.^145^ In another study, Hanna et al. stated that the number of ATMPs approved clinical studies in the EU has increased every year from 12 to 150 numbers between 2004 and 2014. Among the collected data with a total of 939 clinical trials, only 6.9% were in end-stage study (in phase III), which can take another 5 years for commercialization and the remaining 92.2% were in the early stages (phase I, combined phase I, II, and III stages). This may be due to the availability of sponsors (commercial/non-commercial) for the study, lack of market access and launching strategies and financial budget.^146^ Culme-Seymour et al. studied the cost-effective way to deliver engineered trachea for improving the health of three patients (two children and one adult). A patient could undergo the treatment procedure with multiple charges ranging from US$500,000 to US$600,000 based on treatment, availability and follow-up period. It also includes clinical charges (related to the patient’s care at the hospital), clinical testing charges (charges due to the undergone procedures and analysis prior to and after treatment) and charges for engineered air transplant tissue and its maintenance. An estimated total manufacture cost for stem cell engineered trachea replacement is approximately $27,490, which could be considered as a cost-effective treatment in comparison with the quality of life regained after the treatment under severe conditions with available surgical procedures.^147^

## Ethical concerns on TEMPs, 3D printed and bioprinted tissues

### Cell source, processing procedures and their cost

Complex aspects of tissue engineering and bioprinting make its therapeutic potential ethically complicated compared to biologics and drugs.^84,148^ One of the major ethical concerns in TEMPs and bioprinted constructs is the cell source and its processing procedures.^149^ Cells and engineered tissue-based therapies are considered as drugs or biologics in few countries like Japan and the EU union. In these countries, cells and engineered tissue-based therapies are regulated under common legislations. In contrast, cell only therapy (autologous or stem cell) has different regulations when compared to 3D-printed/bioprinted/engineered tissues in US and other countries.^150–152^ The human body has different types of cells such as stem cells, cardiac cells, bone cells, nerve cells, fat cells, blood cells, muscle cells, etc. Among these stem cells, mesenchymal and induced pluripotent stem cells isolated from healthy adult donors are widely used as cell sources in TEMPs and bioprinted constructs due to their high differentiation potential toward any particular lineage of interest and negligible immunogenicity. Further, the use of embryonic stem cells (ESCs) isolated from blastocysts was considered unethical in some countries due to its collection procedure from living or aborted fetuses (either single cell or cluster of cells). These cells should not be intended to use for cloning humans, germ cells, embryos and human-animal chimeras.^153^ However, there may be limitations such as the large risk of patient’s compliance, less therapeutic efficiency after implantation, high cost and compromised safety.^154,155^ There are several successful short-term in vivo studies, but long-term studies have indicated that these cells may lead to the risk of teratoma and cancer related issues.^156^ Numerous studies suggested that MSCs isolated from tissues such as the umbilical cord, bone marrow, adipose tissue, etc., have the potential to differentiate irrepressibly, suppress the immune system and produce new blood vessels, thereby promoting tumor growth and metastasis.^157^ In a study, hMSCs were intravenously injected into female BALB/c 4T1 tumor induced mice model and showed an increase in the tumor growth and metastasis, TGF-beta, interleukins (IL-4, IL-10) and decrease in interferon-gamma production, thereby enhancing the immunosuppressive environment for 35 days.^158^ In the literature, few works reported the use of MSCs, ESCs and iPSCs isolated from mouse, murine, human and chicks as bioink components for the printing of various tissues such as skin, cornea, bone, heart, etc. In many reports, primary cells and other cell types were used for bioprinting applications. For example, Michael et al. have used laser-assisted bioprinter and developed a cellularized skin substitute containing fibroblasts and keratinocytes on Matriderm for full-thickness wounds. The skin substitute was implanted in vivo and showed better integration with the host skin and neovascularization in the affected area within 11 days post-surgery.^159^ In another study, mesenchymal stromal cell lines (D1 cells) along with collagen and nano-hydroxyapatite were bioprinted in situ on mice calvaria defect model and showed enhanced bone volume to total volume using micro-CT experiments after 2 months of treatment.^160^ In conclusion, most of these bioprinted constructs were tested in vivo for its better engraftment with host tissues, vascularization and regeneration efficiency, but these are only initial studies and would have to go a long way through pre-clinical and clinical evaluations.

Patient-specific cells may be another choice to be used as a cell source for both seeding on TEMPs and bioprinting organs to avoid the ethical issues on cell sources. It is possible to isolate stem cells from patients with informed consent, thereby providing an immediate solution for the shortage of organ donors and limiting the use of immunosuppressants.^161^ However, the cost of stem cell collection procedure, in vitro culturing and fabrication procedures would require GMP sophisticated laboratories, expensive consumables, equipment, quality assurance testing, researchers, surgeons and biomedical engineers would be highly expensive and hence only benefits high income communities.^162,163^ Yet, customized and personalized products developed using the bioprinting technique may reduce the cost as they are 3D bioprinted on a very small scale.^2^ On the whole, 3D bioprinted organs would be more beneficial for the recipients waiting for donors. We hope that it may reduce the black market for organs or human transplantable materials if the cost gets reduced.^164^ However, clinical translation of 3D bioprinting may be a huge challenge while considering the social stratification, global inequalities and overall cost.^148^ Further, implementation of this newer technology into the existing healthcare and insurance systems requires careful considerations.

### Variability after implantation

An important facet to be considered after implantation of TEMPs in patients’ body is that it exhibits an inherent variability due to the dynamic response of metabolically active cells in the host ECM environment, which may develop immune rejections or mismatch within the patient’s body.^165^ In some cases, migration of the impregnated cells from TEMPs to other parts of the host body through the bloodstream may occur and this may cause unnecessary adverse events such as cancer or any unknown diseases. Some common ethical issues and risks associated with TEMPs are microbial contamination(re-infection), insufficient sterility and toxicity due to the presence of allogenic sources, dysfunction of bioactive motifs, over reactions of growth factors and toxicity of cryo-preservatives.^166^ Further, clinical trials on 3D bioprinted tissues require a more individual centric approach (transplantation medicine) than conventional normal trials. For example, these products have to be tested in an individual subject and should be continued on other subjects only after evaluating the clinical response in the first subject.^167^ Furthermore, 3D bioprinted tissue/organs could cause a high risk to the subject and hence such trials should be done when there is no alternative option for the chosen subject.

### Ownership of products

Finally, the ownership of products or bioprinted organs would be an issue for the donors, recipients, researchers, doctors and product manufacturing companies. A few known issues are (i) the donors must be informed about the future and further applications of their donated stem cells; (ii) safety issues related to the blueprint of patient’s organ since advancement in genetic engineering may develop a cloned organs or simple biological building blocks (though it may take several decades to commence) and (iii) the owner of developed product for a patent will be an issue either for researcher, patient or company.^165^

## Conclusion

Fabrication of personalized living tissues/organs is now more possible and realistic with the approaches in 3D-bioprinting. In spite of the availability of adaptive regulations, only a very few TEMPs have been licensed. Even though scientific requirements and pre-clinical standards are constantly revised, several tissue engineered products and 3D bioprinted tissues are yet to enter clinical trials. This could be attributed to the fact that the current legislation and standards are more suited for conventional drugs/therapeutics and therefore fails to fully absorb the complexities involved in the latest medicinal products. Hence, it is imperative for the regulatory authorities and other parties to learn the background of these advanced products and adapt the complexities to facilitate the approval processes for TEMPs and 3D bioprinted tissues. Further, the clinicians and researchers should learn the scientific requirements, background of standards, necessary training on clinical trials and GMPs to fulfill the needs of regulatory bodies and global standards. In conclusion, considering the nature of TEMPs and 3D bioprinted tissues, it is essential to make sure that the products are within shelf-life time at the time of clinical procedure and tested with well-planned protocols to assess the safety and long-term efficacy.

## Future perspectives

In order to anticipate the future of 3D bioprinting, it is imperative to understand the current advancements achieved in the successful development of 3D bioprinted tissues such as skin, cartilage and bone. This achievement is due to the clinically most relevant features attained in printing these constructs such as suitable cell types, desired functional aspects, biomimetic resolution and other required associated cues, including vascular network. Therefore, successfully 3D bioprinted skin, cartilage and bone could serve as a yardstick to envision the future prospects and commercialization scope of fabricated constructs.

**Figure 4.**
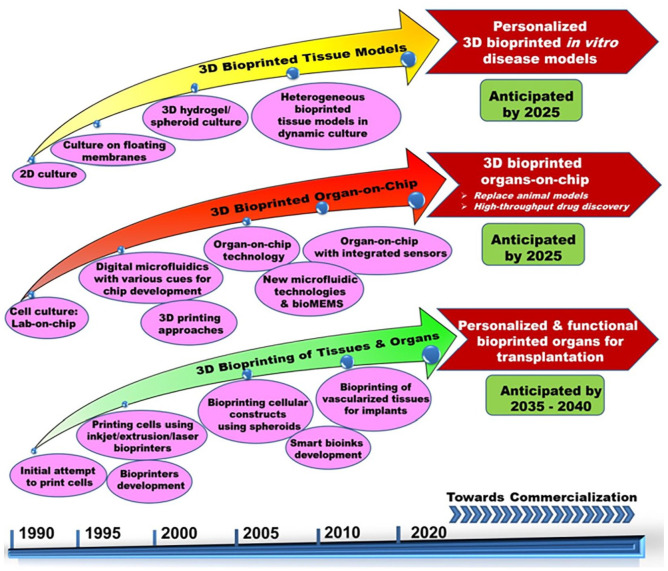
Timeline of significant breakthroughs in 3D bioprinting of organs/tissue models and organ-on-chip development toward effective clinical translation and envisaged commercialization scopes.

Figure 4 shows the 3D bioprinted tissues/organs, and bioprinted organ-on-chip developments along with anticipated years for regulatory approval and commercialization. On considering the medical device, ISO and other regulations in force, we anticipate that bioprinted 3D tissue models and organ-on-chip may not undergo stringent regulations and hence could be envisaged for commercialization possibilities in the next 5–8 years. In contrary, commercialization chances for 3D bioprinted tissues and organs are anticipated only in the forthcoming decades due to complex nature and multiple biological compositions. Additionally, a collaborative approach between academia, industry and hospitals is needed to draw regulatory amendments, which could further improve the clinical translation of bioprinted organs/tissue.

Despite the benefits of creating fully functional soft and hard 3D tissues with neovascularization ability, additional challenges include culturing the printed heterogeneous tissues/organs with a different supporting medium in a bioreactor or with any special set up for maturation. Further, the highest possible printing resolution with a laser bioprinter is 20 µm while small capillaries of native tissues are 3 μm in diameter. In addition to the small diameter, the complexity of vascular networks with the multi-level organization is yet to be achieved in 3D bioprinting. Other challenges are the requirement of a huge number of cells for printing, the need for extensive research on optimal bioink composition with good printing capabilities, shape fidelity and the demand for sophisticated advanced complementary bioprinting technologies with ensured environment and sterility maintenance. Additionally, bioprinting has several other difficulties such as ethical issues, safety, affordability, and large-scale production for successful translation into clinical practice. Marketing approval for the bioprinted tissue equivalents is yet to be achieved with the current/revised legislation and hence focus is needed toward these directions to utilize 3D bioprinting for the fabrication of organs for transplantation needs. As a next-generation fabrication approach, the 4D bioprinting concept with an additional ability of printed constructs to undergo complete maturation in response to dynamic external stimuli may be followed. In the future, several extensive researches on smart bioink composition with excellent shape fidelity, feasibility for large scale production, potential bioprinters and faster clinical trials for the developed bioprinted constructs will definitely pave the way for clinical success and commercialization scope as compared to conventional TEMPs.
